# Upregulation of EFCAB7 after radiofrequency ablation promoting hepatocellular carcinoma metastasis and survival by regulating PARK7

**DOI:** 10.18632/aging.206073

**Published:** 2024-09-12

**Authors:** Dan Cui, Hongye Wang, Zhi Wang, Zhaorong Wu, Min Ding, Linke Bian, Jiachang Chi, Bo Zhai

**Affiliations:** 1 Department of Interventional Oncology, Renji Hospital, School of Medicine, Shanghai Jiao Tong University, Shanghai, China

**Keywords:** EFCAB7, PARK7, radiofrequency ablation, liver cancer

## Abstract

Background: Radiofrequency ablation (RFA) is an established treatment for unresectable and early-stage hepatocellular carcinoma (HCC). However, in some cases, residual tumor cells undergo malignant transformation following RFA. The molecular mechanisms underlying this phenomenon remain poorly understood. EFCAB7, a member of the EF-hand structure family, is of particular interest due to its association with oncogenesis. Nevertheless, the role of EFCAB7 in oncogenesis remains unclear.

Methods: Gene expression level of EFCAB7 in HCC tissues before and after RFA was measured, while *in vitro* and *in vivo* experiments were proposed for exploring the roles of EFCAB7 in tumor cell proliferation and metastasis. Mass spectrometry and CO-IP were adopted to validate the interaction between PARK7 and EFCAB7. Finally, PARK7 in EFCAB7 silencing cells was overexpressed and different functions were measured *in vitro* to determine regulation between two genes.

Results: EFCAB7 showed increased expression after RFA in patient samples and EFCAB7 expression correlated with poor prognosis in HCC patients from the TCGA database. Then, EFCAB7 promoted HCC tumor cell proliferation and metastasis while inhibiting apoptosis. Furthermore, Mass spectrometry and Co-IP experiments revealed a direct interaction between EFCAB7 and PARK7. Finally, when we overexpressed PARK7 in EFCAB7 knockdown tumor cells, it rescued proliferation and metastasis, indicating a functional relationship between these two genes.

Conclusions: EFCAB7 might be a core contributor to HCC cells’ malignant transformation after RFA and could be a potential novel target to provide a therapeutic strategy for the prevention of recurrence after RFA in HCC.

## INTRODUCTION

Hepatocellular carcinoma (HCC) has been one of the most common cancers in Asia and ranked third among cancer related mortality worldwide. Morbidity of this disease has increased in recent years, reaching as high as 523,000 cases in men and 226,000 cases in women annually [1, 2]. Traditional surgical resection has been considered as a gold standard for the treatment of HCC. However, decompensation of liver function may increase the surgery risk. According to the therapeutic consensus of hepatocellular carcinoma, radiofrequency ablation (RFA) has been recommended as an alternative for unresected HCC [3], which facilitates tumor cell death and thermal injury by transferring electromagnetic energy and is broadly applied to the treatment of other cancers in the kidney, lung and bone. Based on results from large multicenter randomized clinical trials, RFA has obtained comparable curative while treating small HCC, compared with surgical resection [4].

Recently, there has been growing evidence indicating that insufficient radiofrequency ablation (iRFA) may facilitate cancer cell progression in clinical practice [5–7]. RFA has been associated with elevated IL-6 and hepatocyte growth factor (HGF)/c-Met Pathway activation [8, 9]. These cytokines and supportive factors facilitate liver regeneration and residue tumor cell growth at the same time. Prevention of residue tumor growth after RFA treatment can improve patients’ prognosis. However, the underlying mechanisms are still waiting for further exploration.

EFCAB7 features an EF-hand structure, which was first proposed by Krestinger while analyzing the three-dimensional structure of parvalbumin by X-ray diffraction [10]. EF-hand structure folds into a special helix-loop-helix structure, containing a highly specific Ca2+ binding site. Many proteins with an EF-hand structure play an important role in cancer and serve as predictive biomarkers and new therapeutic drug targets [11–13]. Although studies have revealed the basic biological function of proteins with an EF-hand structure, the function of EFCAB7 remains a mystery. Only a few studies concerning Ellis-van Creveld syndrome reported the function of EFCAB7 [14]. EFCAB7 was first discovered in the detection of ciliary proteomics, and was then found to be capable of forming a protein complex by combining with IQ-domain containing protein E (IQCE), acting as positive regulators of Hedgehog signaling pathways by interacting with EVC-EVC2 complex [15]. However, the role of EFCAB7 in tumor development and malignancy transformation is still unclear.

In this study, it was found that EFCAB7 is greatly upregulated in HCC tissues after radiofrequency ablation, proving the potential protective role of this protein during intrinsic tumor cell-mediated escape after thermal ablation. After performing *in vivo* and *in vitro* experiments, the knockdown of EFCAB7 was found to greatly dampen HCC cell proliferation and migration while increasing cell apoptosis. Then, interacting proteins with EFCAB7 were discovered by mass spectrometry and it was proven that EFCAB7 is strongly associated with PARK7 and acts as an up-stream molecule upon EFCAB7. Taken together, the present study illustrated one underlying mechanism behind tumor malignancy transformation after insufficient thermal ablation.

## RESULTS

### EFCAB7 was upregulated after radiofrequency ablation and was associated with poor prognosis in liver cancer

**Figure 1:**
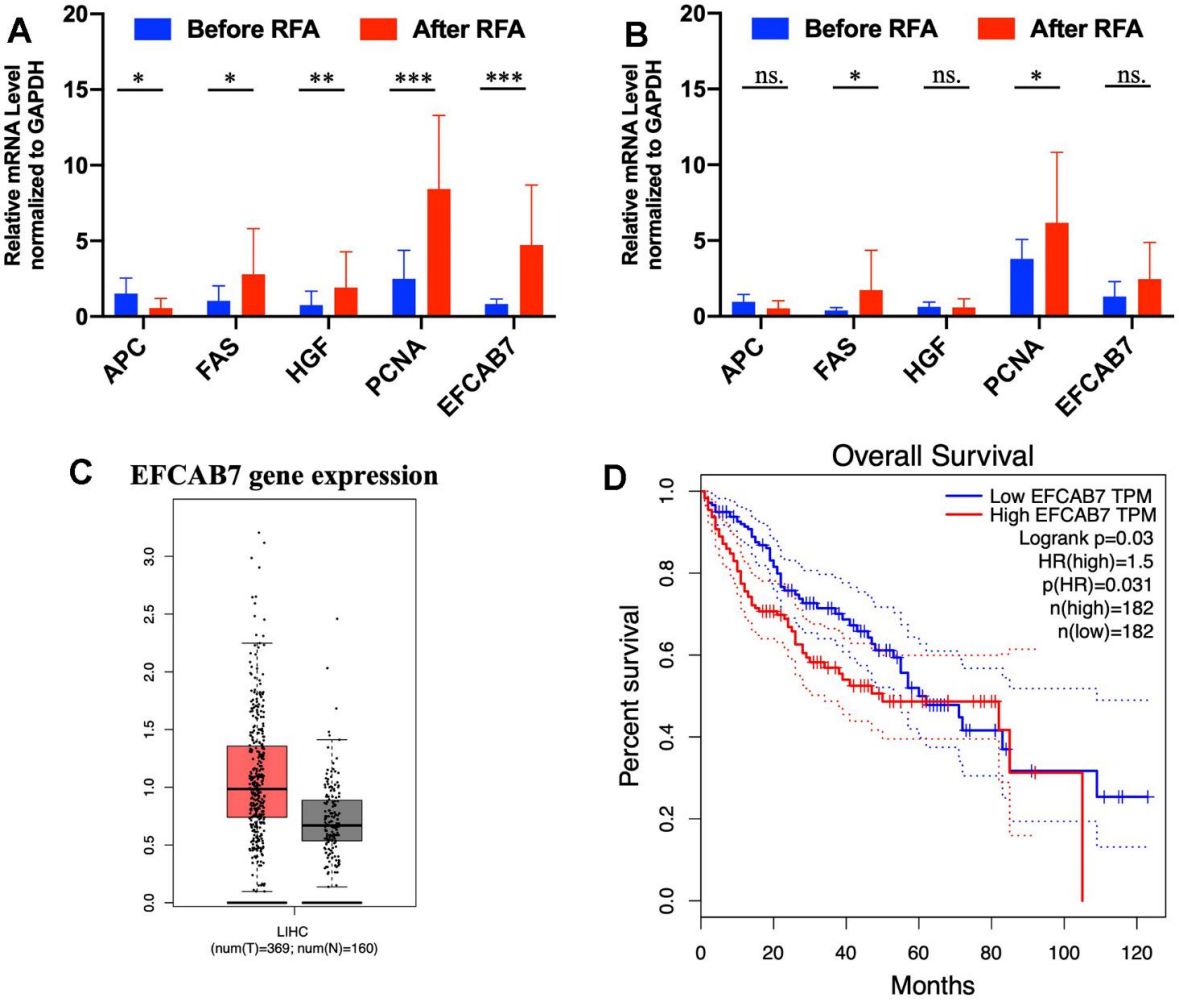
**EFCAB7 was upregulated after RFA and was associated with poor prognosis in HCC.** (**A**, **B**) RT-PCR analysis of *EFCAB7, APC, FAS, HGF* and *PCNA* in HCC tumor tissues and adjacent tissues before and after RFA; (**C**) Expression of *EFCAB7* in TCGA database; (**D**) Kaplan-Meier analysis demonstrated that HCC patients with low *EFCAB7* expression had a better overall survival rate than patients with high *EFCAB7* expression. Student’s t-test and log-rank test (*P < 0.05, **P < 0.01, ***P < 0.001).

Some studies have suggested that inadequate radiofrequency ablation and brief heat exposure may promote tumor metastasis. During this process, there are significant alterations in hepatocyte growth factor (HGF), inflammatory genes, proliferation-related genes, and tumor suppressor genes, all of which contribute to facilitating the growth of residual tumors [5, 16]. Based on these findings, 11 pairs of tumor and adjacent tissues before RFA and after RFA were collected for assessing mRNA expression of *APC, FAS, HGF, PCNA* and *EFCAB7*. In accordance with previous studies, *HGF, PCNA* and *FAS* (P<0.05) were greatly upregulated, with *APC* being downregulated after RFA treatment (Figure 1A). Remarkably, EFCAB7 was significantly upregulated in tumor tissues after radiofrequency ablation (P<0.001, Figure 1A). However, mRNA expression of *APC, HGF, PCNA* and *EFCAB7* in adjacent tissues before and after RFA therapy was found not to be changed significantly (Figure 1B). These results prompted the test over the potential role of EFCAB7 during insufficient RFA-mediated tumor growth. In the TCGA cohort, it was found that EFCAB7 was upregulated in liver cancer tissues compared with normal liver tissues (Figure 1C) and high expression of EFCAB7 was correlated with poor clinical prognosis in HCC patients (P=0.03, Figure 1D), implying that EFCAB7 might function as an oncogene during liver cancer development and protect liver cancer cells from heat injury during RFA.

### EFCAB7 enhanced cell proliferation and inhibited apoptosis *in vitro* and *in vivo*

**Figure 2:**
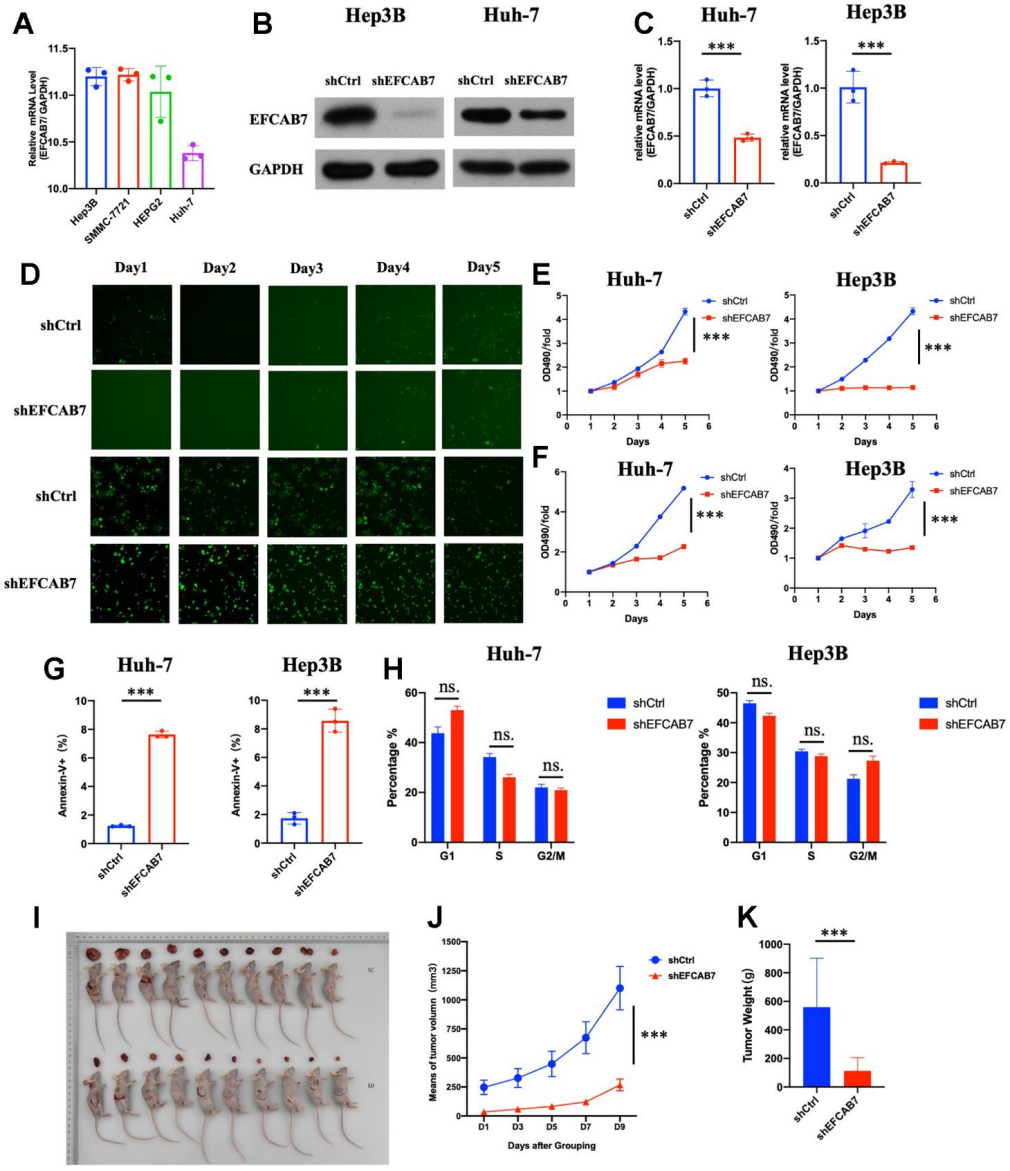
**EFCAB7 enhanced HCC proliferation and inhibited apoptosis *in vitro* and *in vivo*.** (**A**) RT-PCR analysis of *EFCAB7* in four different HCC cell lines; (**B**, **C**) Western blot and qPCR detecting knockdown efficiency of EFCAB7 in Huh7 and Hep3B HCC cell line; (**D**) Representative images of Huh7 and Hep3B after EFCAB7 silencing in the same fields after 1, 2, 3, 4 and 5 days; (**E**) Tumor cell proliferation was detected by CCK-8 assays after 1, 2, 3, 4 and 5 days and normalized to D1. (n = 3); (**F**) Proliferation of stable silencing Hep3B and Huh7 cells was evaluated by MTT assay in 1, 2, 3, 4 and 5 days. (n=3); (**G**) Apoptosis rate of tumor cells after knocking down EFCAB7; (**H**) Cell cycle analysis after knocking down EFCAB7; (**I**) Hep3B tumor cells (4 × 10^6^ cells per mouse, n = 10 for each group) were subcutaneously inoculated in BALB/c nude mice. The mice were sacrificed at 10 days. (**J**) The tumor volumes were also measured in different days after inoculation; (**K**) Tumor weight was measured at 10 days. one-way ANOVA and Student’s t-test were used to compare differences with continuous variables (*P < 0.05, **P < 0.01, ***P < 0.001).

mRNA expression of EFCAB7 was compared in different liver cancer cell lines. Among these cell lines, Hep3B and SMMC-7991 showed higher expression, while Huh7 exhibited lower expression of EFCAB7 (Figure 2A). To determine the function of EFCAB7 in liver cancer, EFCAB7 in Hep3B and Huh7 was knocked down, respectively, and shRNA targeting EFCAB7 demonstrated high silencing efficiency in Hep3B and Huh7 (Figure 2B, 2C). Compared with the control group, silencing EFCAB7 significantly suppressed Hep3B and Huh7 cell proliferation while promoting cell apoptosis (Figure 2D–2G). Considering the deficit of cell growth after EFCAB7 knockdown, cell cycle changes between shCtrl and shEFCAB7 groups in Huh7 and Hep3B cell lines were compared, respectively, with only minimal changes observed in the G1 and G2/M phases after silencing EFCAB7 (Figure 2H).

EFCAB7 was observed to influence tumor cell growth *in vitro*. Therefore, attempts were made to determine the function of EFCAB7 *in vivo*. shEFCAB7 and shCtrl Hep3B liver cancer cells were implanted into nude mice. Similar to the findings *in vitro*, groups silencing EFCAB7 markedly inhibited tumor cell growth *in vivo* (Figure 2E–2G). Collectively, these results confirmed that EFCAB7 promotes HCC tumorigenesis both *in vitro* and *in vivo*.

### EFCAB7 played an important role in liver tumor cell metastasis

**Figure 3:**
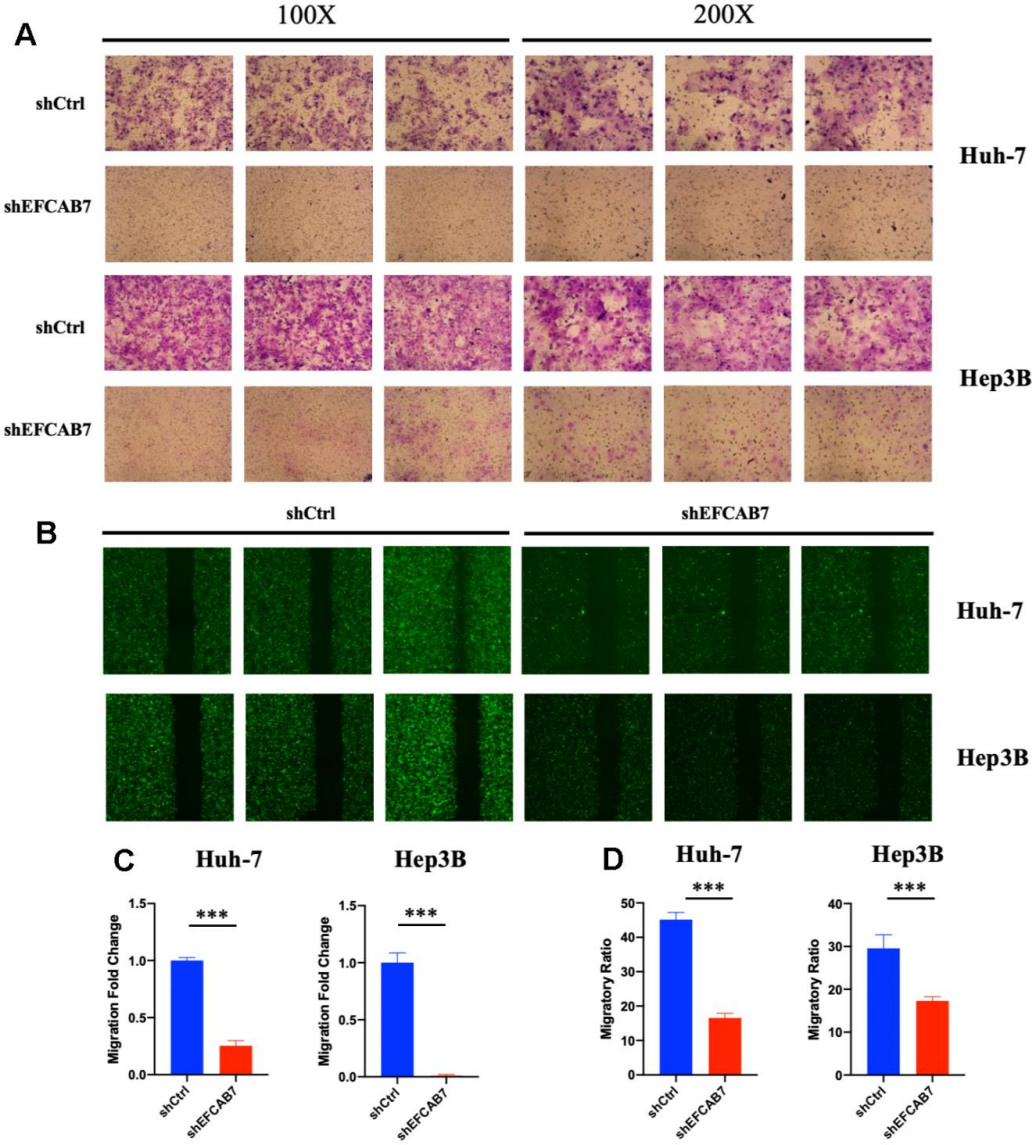
**EFCAB7 promoted Hep3B and Huh7 cells metastasis *in vitro*.** (**A**) Transwell assays were used to assess the influence of EFCAB7 on the invasion abilities of stable Hep3B and Huh7 cells; three representative images under 100X and 200X are presented. (**B**) Wound healing assays were performed to detect the migration abilities of Hep3B and Huh7 cells; (**C**) Migratory fold change was measured based on Transwell assays; (**D**) Migratory ratio was measured based on wound healing assays. Student’s t-test (*P < 0.05, **P < 0.01, ***P < 0.001).

Given that EFCAB7 is significantly upregulated after RFA and our previous results have shown that EFCAB7 promotes liver cell growth, we further investigated whether EFCAB7 could also enhance tumor cell metastasis. To confirm this hypothesis, we compared the cell migration capacity of tumor cells between the EFCAB7 knockdown groups and the control groups using Transwell Assay (Figure 3A, 3C). The results clearly indicated that tumor cells lacking EFCAB7 had a significantly reduced ability to invade. Consistent with the Transwell Assay results, data from the wound healing assay also confirmed the crucial role of EFCAB7 in tumor cell metastasis (Figure 3B, 3D).

To date, EFCAB7 was found to serve as an oncogene and might influence tumor cell proliferation, apoptosis, and metastasis.

### EFCAB7 interacted with PARK7 and regulated many biological processes

**Figure 4:**
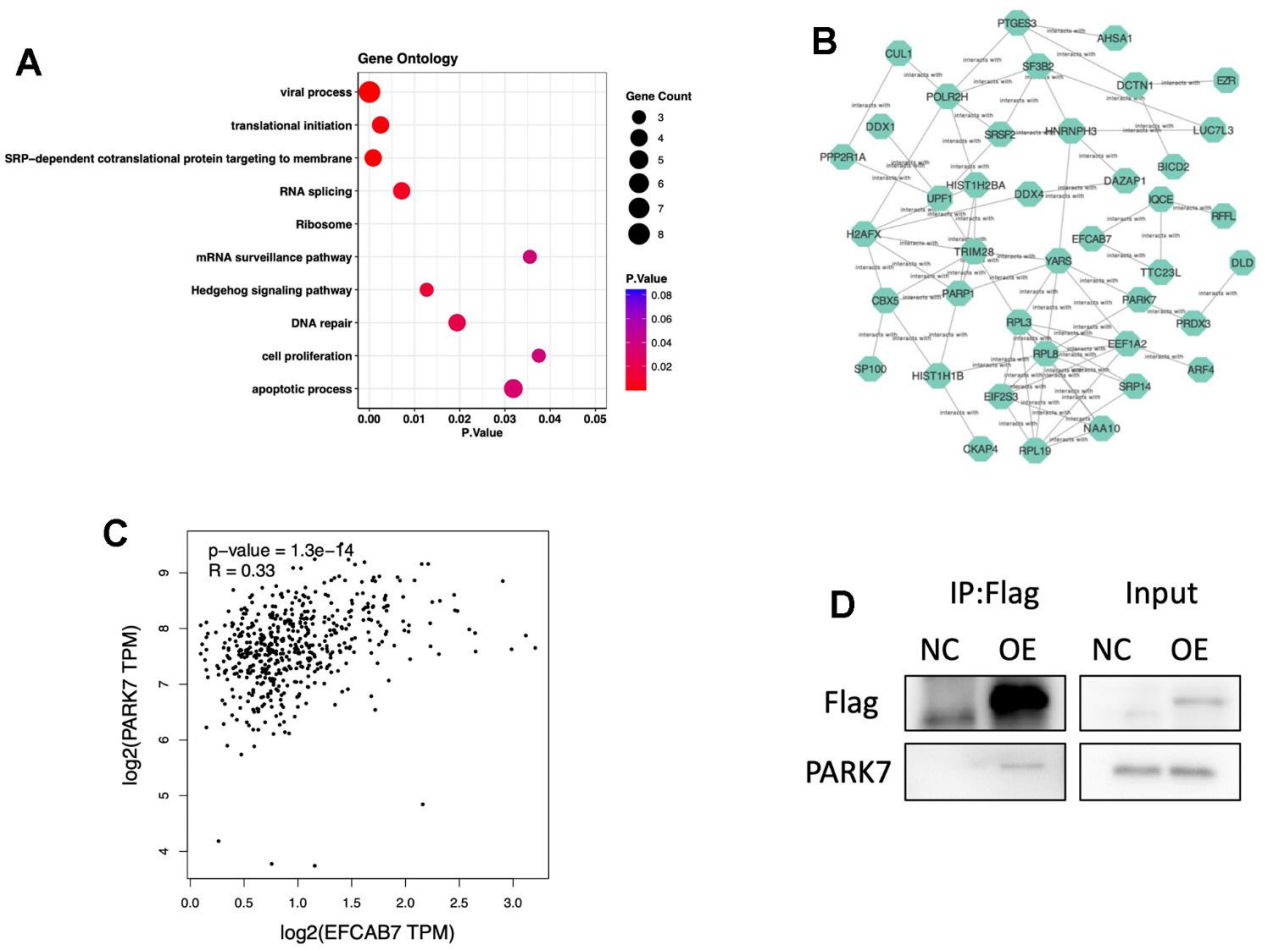
**EFCAB7 might participate in many biological processes and interact with PARK7.** (**A**) Gene Ontology of proteins directly interacting with EFCAB7 via mass spectrometry; (**B**) Gene co-expression network of EFCAB7 interacted proteins; (**C**) Correlation of PARK7 and EFCAB7 gene expression level in TCGA LIHC cohort; (**D**) Co-IP demonstrating PARK7 directly interacting with EFCAB7 in Hep3B. Student’s t-test and Pearson’s test (*P < 0.05, **P < 0.01, ***P < 0.001).

All the results strongly suggest that EFCAB7 plays a pivotal role in liver tumor cell proliferation, apoptosis, and metastasis, prompting further exploration of the specific molecular mechanisms involving EFCAB7. Consequently, we employed mass spectrometry to identify proteins that interact with EFCAB7, resulting in the identification of 48 potential interacting proteins. Gene ontology analysis revealed that EFCAB7 and its associated interacting proteins are involved in several critical biological processes, including translation initiation, viral processes, the hedgehog signaling pathway, DNA repair, cell proliferation, and apoptosis (Figure 4A). Furthermore, co-expression network analysis confirmed the coordinated expression of these genes (Figure 4B). Notably, among these candidate molecules, the gene expression level of PARK7 was found to be highly correlated with EFCAB7 based on TCGA cohort data (Figure 4C). Subsequently, we conducted CO-IP experiments, which confirmed that EFCAB7 directly binds to PARK7 in liver cancer cells, consistent with the mass spectrometry results (Figure 4D). In summary, these findings collectively demonstrate that EFCAB7 can form complexes with PARK7 and regulate various crucial biological processes during oncogenesis.

### PARK7 was a downstream molecule under EFCAB7 and the overexpression of PARK7 could rescue EFCAB7 deficiency

**Figure 5:**
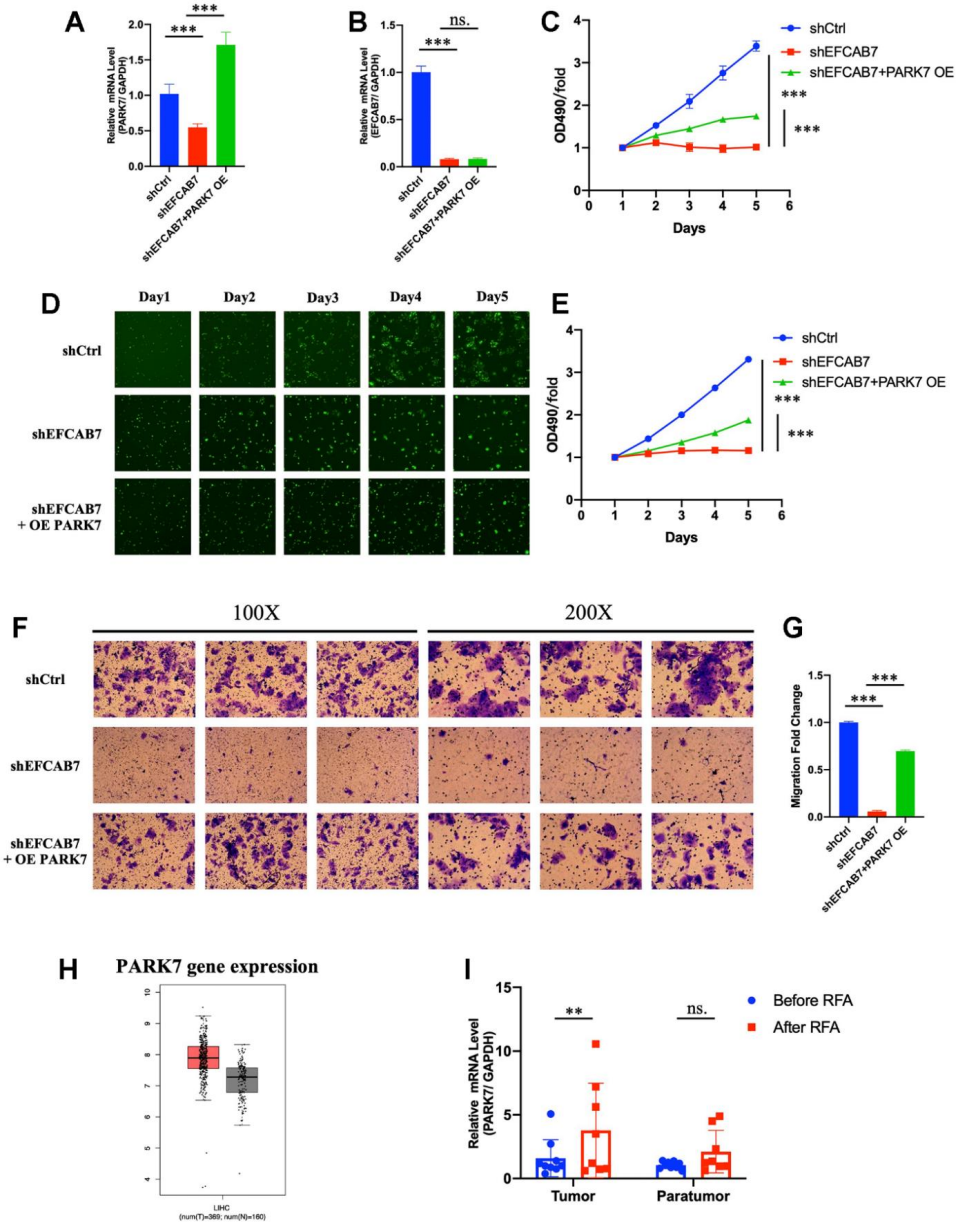
**PARK7 functioned as a downstream molecule under EFCAB7.** (**A**). RT-PCR analysis of the overexpression efficiency of PARK7 in EFCAB7 knockdown Hep3B cell line; (**B**). RT-PCR analysis of EFCAB7 mRNA expression level after the overexpression of PARK7 in Hep3B cell line; (**C**). Proliferation of stable transfected Hep3B tumor cells was measured by absolute cell counting (n=3); (**D**). Representative images of treated Hep3B in the same fields after 1, 2, 3, 4 and 5 days. (**E**). MTT assay analyzed the growth of stable transfected Hep3B tumor cells in different groups; (**F**) and (**G**). Transwell assays were conducted to measure the migration abilities of different treated tumor cells; (**H**). Expression of PARK7 in TCGA database; (**I**). RT-PCR analysis of PARK7 in HCC tumor tissues and adjacent tissues before and after RFA. Student’s t-test (*P < 0.05, **P < 0.01, ***P < 0.001).

Since PARK7 was found to directly interact with EFCAB7, we conducted further investigations to determine whether PARK7 operates as a downstream molecule regulated by EFCAB7. To test this hypothesis, we initially assessed the gene expression of PARK7 in an EFCAB7 knockdown cell line using RT-PCR and observed a significant downregulation of PARK7 (Figure 5A). Subsequently, we overexpressed PARK7 in the EFCAB7 knockdown cell line and examined EFCAB7 expression (Figure 5A). Importantly, overexpression of PARK7 did not alter the gene expression of EFCAB7 (Figure 5B), suggesting that PARK7 likely functions downstream of EFCAB7.

We then explored whether the overexpression of PARK7 could rescue the growth defects induced by EFCAB7 silencing. Intriguingly, PARK7 overexpression substantially increased cell growth in EFCAB7 knockdown tumor cells (Figure 5C–5E). Furthermore, we found that PARK7 overexpression enhanced cell migration in EFCAB7 knockdown tumor cells (Figure 5F, 5G). In a clinical context, analysis of data from the TCGA database revealed that PARK7 was significantly more highly expressed in tumor tissues compared to adjacent non-tumor tissues (Figure 5H). Additionally, PARK7 expression was found to be increased in tumor tissues after RFA treatment (Figure 5I). Collectively, these results provide compelling evidence that EFCAB7 regulates cell growth and migration through its interaction with PARK7.

## DISCUSSION

Growing evidence suggests that incomplete radiofrequency ablation (iRFA) may lead to malignant transformation in liver cancer. One of the primary characteristics of residual hepatocellular carcinoma (HCC) after insufficient RFA is increased tumor cell proliferation and resistance to chemotherapy. This study demonstrates that sorafenib effectively inhibits the rapid growth of residual cells following insufficient RFA by targeting the hypoxia-inducible factor-1α (HIF-1α)/VEGFA pathway. Additionally, silencing HIF-1α has been shown to reduce the invasion and metastasis of HCC. These findings shed light on potential therapeutic strategies for managing HCC after insufficient RFA [17]. Furthermore, it was reported in an independent study that insufficient RFA promoted SMMC7721 cell proliferation by activating CaMK II/ERK-dependent overexpression of VEGF [18]. Although some studies have explored the mechanisms and prevention strategies of HCC progression after insufficient RFA, specific molecular mechanisms are still waiting to be explored.

In this study, EFCAB7 was found to be dramatically upregulated after radiofrequency ablation in patient samples. In the TCGA database, EFCAB7 was highly expressed in HCC tissues than in para-tumor tissues. Additionally, the Kaplan-Meier analysis found that high EFCAB7 expression was correlated with poor overall survival in HCC patients. It was found by conducting different *in vitro* and *in vivo* experiments that EFCAB7 supported HCC cell proliferation and metastasis while inhibiting apoptosis. All these results indicated that EFCAB7 functioned as an oncogene in HCC and that the upregulation of EFCAB7 after RFA could help HCC reinvigorate. The function of EFCAB7 is revealed for the first time. Then, many proteins were found to interact with EFACB7 in HCC tumor cell lines via mass spectrometry and bioinformatics analyses indicating that EFCAB7 participated in many vital biological processes during tumor development. Specifically, it was testified that EFCAB7 was directly bound to PARK7 and worked as an upstream molecule upon PARK7.

The human PARK7 protein, also known as DJ-1, is a ubiquitously expressed small protein consisting of 189 amino acids as a member of the large PARK7/PfpI superfamily [19]. PARK7 is a multifunctional protein that protects neurons from oxidative stress and is associated with Parkinson’s disease [20], which has been proven to be overexpressed in a variety of tumors, including uveal melanoma, non-small cell lung cancer, hepatocellular carcinoma, pancreatic ductal adenocarcinoma, ovarian cancer, breast cancer, and esophageal squamous cell carcinoma. In some cancers, such as endometrial cancer, non-small cell lung cancer, pancreatic cancer, esophageal squamous cell carcinoma, and cervical cancer, high expression of PARK7 is significantly associated with metastasis or worse prognosis [21]. Accumulated evidence suggests that PARK7 can promote cancer cell death, survival, proliferation and metastasis by regulating redox balance, activating Akt/mTOR, MEK/ERK, NF-κB, and HIFα signaling pathways, or inhibiting p53, JNK, and ASK1 signaling pathways [22]. Recent studies have also confirmed that PARK7 promotes the proliferation and metastasis of colorectal cancer by activating the Hh and Wnt signaling pathways, and that PARK7 increases proteins involved in the Hh signaling pathway, including GLI1, GLI2, and PTCH1 [23]. It is the first study reporting that PARK7 can be directly bound to EFCAB7 and together promote oncogenesis. However, whether EFCAB7 regulates PARK7 protein modification and degradation is still not revealed by existing data. Given that PARK7 is a key regulator in Hedgehog signaling pathway and EFCAB7 can also act as a positive regulator of Hedgehog signaling pathway, it can be reasonably concluded that EFCAB7 and PARK7 could interact together to regulate the Hedgehog signaling pathway, thus promoting oncogenesis. GO analysis in the present study also proves that EFCAB7 participates in Hedgehog pathway. However, there are still many works to be carried out to discover specific mechanisms underlying gene regulation and signaling pathways.

## CONCLUSIONS

In summary, the present study firstly: (1) proposed increasing EFCAB7 expression after radiofrequency ablation; (2) found that EFCAB7 acted as an oncogene and promoted HCC tumorigenesis and progression; (3) claimed that PARK7 was a downstream molecule under EFCAB7, and these two proteins are directly bound together.

## MATERIALS AND METHODS

### Cell culture

Established human liver cancer cells Hep3B, SMMC-7221, HEPG2, Huh7 were obtained from the American Type Culture Collection (ATCC; Manassas, VA, USA). All cell lines in this study were mycoplasma-free. Tumor cells were cultured in a 5% CO2 atmosphere at 37°C and maintained in DMEM medium (Meilunbio, Cat# MA0212) supplemented with 10% fetal bovine serum (Gibco, Cat# 10099-141) and 1% penicillin/streptomycin (Gibco, Cat# 15070063). All cell lines in this study were authenticated using short tandem repeats analysis by Shanghai Biowing Applied Biotechnology (Shanghai, China).

### Patient samples

HCC tissues and paired adjacent tissues were obtained from 11 patients before and after RFA treatment at Renji Hospital, School of Medicine, Shanghai Jiao Tong University from January 2019 to September 2021. HCC tissues were stored at -80°C in Trizol after surgery for RNA extraction. HCC tumor samples were confirmed by two pathologists.

### Lentivirus construction and transfection

For silencing EFCAB7 and overexpressing PARK7 in Huh7 and Hep3B, lentivirus was purchased from GeneChem (Shanghai, China). After transfection, 5μg/ml puromycin (Sigma-Aldrich, St. Louis, MO, USA) was used for selecting stably transfected cells. The EFCAB7 knockdown or PARK7 overexpression were confirmed by immunoblotting and RT-PCR analysis.

### Cell viability assay

To assess cell proliferation and cell viability, we used the Cell Counting Kit-8 (CCK-8) following the manufacturer’s instructions (Dojindo Laboratories, Japan). Treated Huh7 or Hep3B cells were initially seeded at a density of 1000 cells per well in 96-well plates and cultured in 5% CO2 atmosphere at 37°C in the humidified incubator. After several days of culturing, we added 10 μl of CCK-8 solution to each well, and the resulting color was detected at 450 nm using a microplate absorbance reader (Bio-Tek, Santa Clara, CA, USA). Each experiment was conducted in triplicate.

### Invasion and migration assay

The invasion and migration capability of tumor cells were measured via Transwell assay. Matrigel was coated for 30 min at 37°C in a 24-well Transwell chamber. Next, 400 μl of transfected cells suspended in serum-free medium (2 × 10^3^ cells/ml) and 800 μl of culture medium were seeded to the upper and the lower chamber, respectively. After 24h, cells in the lower chamber were fixed with 4% paraformaldehyde for 30 min and stained with crystal violet for 10 min. Images were captured in at least five random fields under a microscope, and these experiments were also performed in triplicate. For wound healing assay, treated Huh7 or Hep3B cells (2 × 10^5^ cells/well) were seeded into 6-well plates and incubated overnight in 5% CO2 atmosphere at 37°C. Then, a straight scratch was created in the center of each well and obtained using a pipette tip. Images of the same location were taken at 24 and 48 hours and normalized to the length at 0 hours. Each experiment was performed in triplicate.

### Real-time quantitative PCR

In accordance with instructions provided by the manufacturer, total RNA was isolated from tumor tissues and cultured cells using Trizol mRNA extracting Kit (Pufei, Shanghai, Cat. No. 3101-100). The purity and concentration of extracted mRNA were examined by strict quality control. Then, 1mg of mRNA was reversely transcribed into cDNA under standard conditions for the commercial Promega M-MLV Reagent Kit, and real-time quantitative PCR was performed with SYBR Premix Ex Taq (Takara, Japan). The cycling condition was set as: 95°C for 30s, followed by 40 cycles of 95°C for 4s and 60°C for 30s. All gene expression results were normalized to GAPDH, and specific gene primers were purchased from GeneCopoeia (Rockville, MD, USA).

### Flow cytometry

The cell cycle and apoptosis of tumor cells were analyzed via flow cytometry. Cell cycle: Fresh cultured tumor cells were fixed in 75% pre-cooled ethanol at 4°C overnight and centrifuged at 1000 rpm. The cells were stained with 50 mg/mL propidium iodide and 100 g/mL DNase-free RNase A for 30 min after being washed with PBS three times and analyzed via flow cytometry. Apoptosis: The apoptosis of transfected tumor cells was assessed according to fluorescence intensity of Annexin-V and PI. Treated cells were washed with PBS twice and re-suspended in 200 μl binding buffer containing 5 μL annexin and 2μl propidium iodide for 20 min in the darkness at room temperature. After incubation, 200μl binding buffer was added to terminate the staining. The apoptosis rate was measured with flow cytometry (BD Biosciences, Franklin Lakes, NJ, USA).

### Co-immunoprecipitation

Co-immunoprecipitation was performed as described previously. Treated cells were washed with PBS twice and were lysed in IP buffer containing 10 mM phosphate buffer, 120 mM NaCl, 2.7 mM KCl, 1% Nonidet P-40, 0.5% DOC, 0.1% SDS supplemented with protease inhibitor mixture (Cell Signaling Technology, Danvers, MA, USA) and phosphatase inhibitor mixture cocktails (Sigma-Aldrich, St. Louis, MO, USA). Co-immunoprecipitations were conducted with 2mg total cell lysates and anti-EFCAB7 antibody on protein A/G mix beads (Thermo Fisher Scientific, Waltham, MA, USA) overnight. Antibody-protein complex was utilized for further immunoblotting.

### Mouse xenograft assay

4–6 weeks old male BALB/c nu/nu mice were purchased from Vital River Laboratory Animal Technology Co., Ltd. (Beijing, China) and raised under standard pathogen-free conditions. Treated Huh7 and Hep3B cells (4 ×10^6^ /per mice) were suspended in 200 μl PBS and then subcutaneously injected into the flanks of BALB/c nu/nu mice (ten mice per group). Tumor volume was serially assessed with calipers (tumor volume = ½ length^2^ × width). All animal experiments were approved by the Ethics Committee for Laboratory Animals of the Affiliated Renji Hospital, School of Medicine, Shanghai Jiao Tong University.

### Statistical analysis

All experiments were conducted in triplicate. The data were analyzed via Student’s t-test or the ANOVA test. Date analyses were performed using GraphPad Prism 8.0 (GraphPad Software, La Jolla, CA, USA). This test was two-sided and P < 0.05 was considered statistically significant.
